# The off-target effects of AID in carcinogenesis

**DOI:** 10.3389/fimmu.2023.1221528

**Published:** 2023-08-04

**Authors:** Junna Jiao, Zhuangwei Lv, Yurong Wang, Liye Fan, Angang Yang

**Affiliations:** ^1^ School of Basic Medical Sciences, Xinxiang Medical University, Xinxiang, Henan, China; ^2^ School of Forensic Medicine, Xinxiang Medical University, Xinxiang, Henan, China; ^3^ Henan Key Laboratory of Immunology and Targeted Therapy, School of Laboratory Medicine, Xinxiang Medical University, Xinxiang, Henan, China; ^4^ The State Key Laboratory of Cancer Biology, Department of Biochemistry and Molecular Biology, Fourth Military Medical University, Xi'an, Shaanxi, China; ^5^ Henan Collaborative Innovation Center of Molecular Diagnosis and Laboratory Medicine, School of Laboratory Medicine, Xinxiang Medical University, Xinxiang, Henan, China

**Keywords:** activation-induced cytidine deaminase, off-target, epigenetic modification, transcriptional regulation, carcinogenesis

## Abstract

Activation-induced cytidine deaminase (AID) plays a crucial role in promoting B cell diversification through somatic hypermutation (SHM) and class switch recombination (CSR). While AID is primarily associated with the physiological function of humoral immune response, it has also been linked to the initiation and progression of lymphomas. Abnormalities in AID have been shown to disrupt gene networks and signaling pathways in both B-cell and T-cell lineage lymphoblastic leukemia, although the full extent of its role in carcinogenesis remains unclear. This review proposes an alternative role for AID and explores its off-target effects in regulating tumorigenesis. In this review, we first provide an overview of the physiological function of AID and its regulation. AID plays a crucial role in promoting B cell diversification through SHM and CSR. We then discuss the off-target effects of AID, which includes inducing mutations of non-Igs, epigenetic modification, and the alternative role as a cofactor. We also explore the networks that keep AID in line. Furthermore, we summarize the off-target effects of AID in autoimmune diseases and hematological neoplasms. Finally, we assess the off-target effects of AID in solid tumors. The primary focus of this review is to understand how and when AID targets specific gene loci and how this affects carcinogenesis. Overall, this review aims to provide a comprehensive understanding of the physiological and off-target effects of AID, which will contribute to the development of novel therapeutic strategies for autoimmune diseases, hematological neoplasms, and solid tumors.

## 1 Introduction

The human body is constantly challenged by various antigens, and antibodies play a crucial role in protecting it from infections caused by these antigens. The generation of antibodies is dependent on B cell differentiation, which undergoes somatic hypermutation (SHM) and class switch recombination (CSR) after V(D)J recombination of immunoglobulins (Igs) genes, then naive B cells mature into functioning B cells. Activation induced cytidine deaminase (AID) is an essential enzyme that induces SHM and CSR by generating the U:G mismatch, converting cytosine (C) to uracil (U) (1–10). AID’s deamination in the process of SHM induces point mutations in the Ig gene, enhancing Ig diversity (11–16), while in the process of CSR, AID drives class switching of IgM to IgG, IgD, IgE, and IgA (3–10).

Apart from AID’s natural physiological function in B cell maturation and antibody production, AID has been identified as an oncogene (17). Due to its intrinsic DNA modifying ability, AID poses a clear threat to genomic stability (17, 18), with off-target modifications predisposing cells to malignant transformation (19–21). This review provides a general overview of AID’s physiological and pathological functions, including its alternative role as a cofactor, with particular emphasis on AID’s off-target effect.

## 2 Biological functions of AID

**Figure 1:**
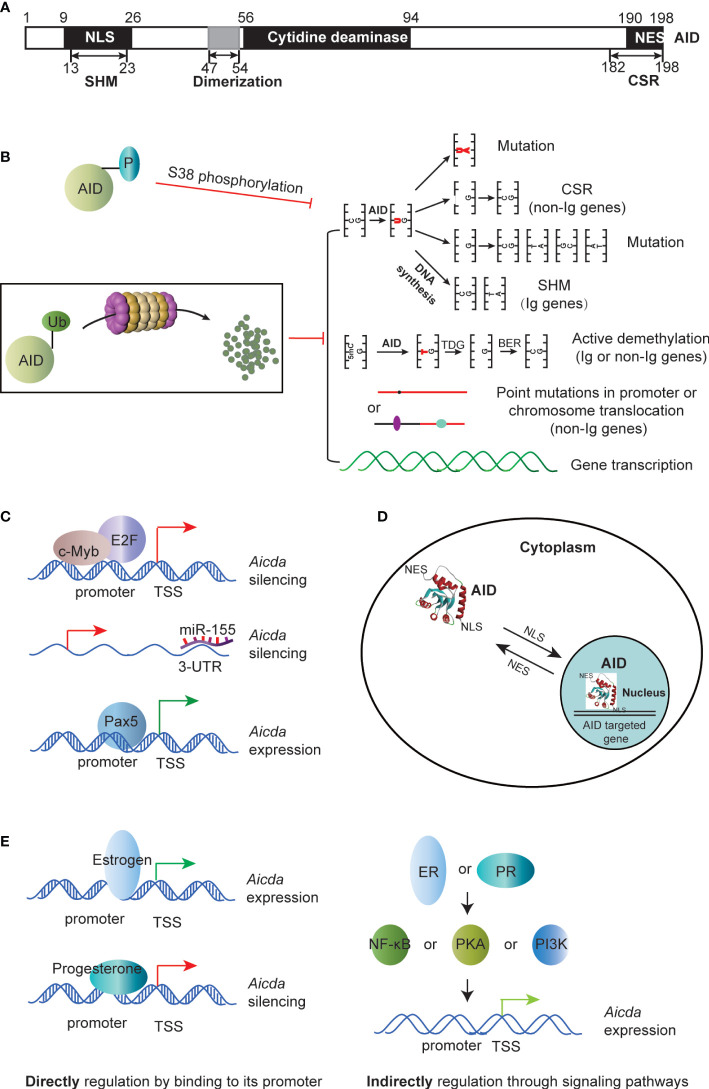
AID’s function and regulation of AID. **(A)** The primary structure of AID. **(B)** The phosphorylation and ubiquitination of AID control AID’s function. AID’s function includes (1): AID mediates CSR and SHM to Ig genes (2); AID induces active methylation to both Ig and non-Ig genes (3); AID causes point mutations or chromosome translocation to non-Ig genes (4); AID modulates gene transcription as a cofactor. **(C)** Regulation of AID by transcription factors, such as c-Myb and Pax5, and miRNAs such as miR-155. They directly binding to the promoter of Aicda. **(D)** The NLS and NES in the structure of AID control the life span of AID in nucleus, and mediate the contact between AID and genomic DNA. **(E)** Hormone (such as estrogen and progesterone) activates or inhibits AID by directly binding to its promoter or indirectly through signaling pathways, such as NF-κB, PKA and PI3K. NLS, nuclear localization sequence; NES, nuclear export signal.

Through the extraction of cDNAs from both switch-induced and uninduced murine B lymphoma CH12F3-2 cells, a novel enzyme, AID, encoded by the AICDA gene has been identified. AID is homologous to the mRNA-editing enzyme, catalytic polypeptide 1 (APOBEC-1) (1–3). *AICDA* is located on human chromosome 12 and encodes 198 amino acids. The catalytic center for cytosine deaminase and the protein-like domain of AID’s structure are similar to APOBEC1, thus AID could recognize a specific RGYW/WRCY motif (R=A/G, Y=C/T, W=A/T) and deaminate cytidines (C) to uracil (U), resulting in a U:G mismatch (3–10) (**Figure 1A**).

### 2.1 AID and SHM

SHM primarily occurs in the variable region genes of IgH and IgL. The mutation sites usually happen in the C of the mutation hotspot WRCY, which resides within 1.5 kb downstream of the transcription start site (TSS) (3, 11). Prior to SHM, single-strand DNA transcription vesicles form on the Ig v;ariable region during transcription, producing single strand DNA (ssDNA). AID targets the cytosine of ssDNA and converts C to U, creating a U:G mismatch. The cell then selects one of three ways to repair the U:G mismatch: DNA replication, base excision repair (BER), or mismatch repair (MMR) (12). DNA replication repair leads to C-T mutations in the AID-targeted DNA stand and G-A mutations in the complementary strand (13, 14). In BER, the UNG removes the uracil residue to create a gap, which is then converted into a single strand break (SSB) by APE. The low-fidelity DNA polymerase randomly inserts any one of the four bases A, T, C or G at the SSB, causing mutations (15). In MMR, the U:G mismatches are recognized by Msh2-Msh3 or Msh2-Msh6 heterodimers, and MLH1 and PMS2 are recruited to produce SSB, and exonuclease 1 removes a series of nucleotides near the mismatches, then proliferating cell nuclear antigen (PCNA) recruits the low-fidelity error-prone DNA polymerase to repair the gap, resulting in multiple base mutations to replace the original U:G mismatch site (**Figure 1B**) (16).

### 2.2 AID and CSR

The C-region exon of each IgH contains a switch region (S region) that is composed of highly repetitive sequences rich in the WRCY motif. Upstream of this region, there is an intron (region I) and its promoter. Upon stimulation with antigen and cytokines, transcription of the germline IgH begins from the activated region I promoter and produces the I_H_-C_H_ transcript (3–5). It is currently believed that transcription of the I_H_-C_H_ results in accessibility to the S region, which causes the S region DNA to uncoil and form an open structure. This state promotes AID to target ssDNA of the S region sequence. During the class switch from IgM to IgE, transcription of the Ig Iϵ-Cϵ germline occurs, which results in AID deaminating the cytosine in the ssDNA of the S region and converting it into uracil, leading to U:G mismatches (6). This results in the formation of a SSB which is facilitated by the uracil-DNA N-glycosidase (UNG) and apurinic/apyrimidinic (AP) endonuclease (APE) (5, 7). As a result, a double-strand break (DSB) is formed in the Sμ and Sϵ regions, and repair is carried out by the canonical non-homologous end-joining (cNHEJ) pathway (5, 8, 9). The KU70 and KU80 proteins form a heterodimer to recognize and bind to the DSB terminus, while DNA-PKcs and Artemis process the DNA terminus. Next, X-ray repair cross-complementing protein 4 (XRCC4) and DNA Ligase IV, together with XRCC4-like factor (XLF), complete the VDJ-Cϵ splicing process to generate the Sϵ-Sμ looped out sequence. The final VDJ-Cϵ sequence is a complete Ig heavy chain gene. When the core component of the cNHEJ pathway is missing or insufficient, the alternative NHEJ (aNHEJ) pathway repairs the DSB, which has a high error rate (9). The aNHEJ pathway can easily cause genetic mutations or chromosomal translocations that eventually lead to cell death or cancers (10) (**Figure 1B**).

## 3 AID’s off-target function

### 3.1 AID’s mutation function

It is known that both SHM and CSR are linked to transcription. The key aspects of this association can be summed up as follows: firstly, SHM and CSR are crucial for the transcription of variable and switch regions, respectively, and mutations in Ig promoters have a significant impact on the frequency of SHM and CSR; secondly, the accumulation of mutations commences 100 bp downstream of the TSS in Ig genes and peaks at 200 bp downstream, subsequently decreasing to undetectable levels over the next 1.5-2 Kb. This specific mutational characteristic is related to the accessibility of AID to Ig genes; thirdly, AID is capable of recognizing and deaminating C in ssDNA instead of double-stranded DNA (dsDNA) *in vitro* (4, 5, 12–16). Moreover, as AID targets non-Ig genes due to the non-conserved nature of its targeted hotspot WRCY, it can result in promoter mutations or chromosome translocations in non-Ig genes, leading to off-target functions of AID (17, 19–22). This off-target mechanism frequently results in abnormal transcription of numerous cancer-associated genes (17, 19–22).

The genes mutated by AID possess a transcriptional landscape characterized by super-enhancers and convergent transcription (17). This suggests that AID targets the transcriptional activation region of local genes (23, 24), which corresponds to AID’s accessibility to ssDNA that is typically present in highly transcribed regions like promoters, enhancers, and super-enhancers. This chromatin architecture can be explained by two points: (i) AID targets non-conservative “hot spot motifs” WRCY, which are rich in C and G, and CpG islands that are abundant in promoters and super-enhancers, indicating AID’s mutation function in non-Ig gene promoters and super-enhancers. (ii) AID occupies sufficient space on non-Ig genes, indicating its accessibility to non-Ig genes (19). The transcriptional frame accessible to AID targeting has been noted to be established by recruiting RNA PolII, Spt5, and RPA (25, 26). AID mediates the mutation of genes involved in B-cell transformation and several hematopoietic neoplasms with the help of these transcription factors. Examples of these genes include *BCL6*, *RHOH*, *PIM1*, *EBF1*, *PAX5*, *MYC*, *PTEN*, *GATA3*, *TCF-7*, *BCL2*, *BTG2*, *CXCR4*, *ZFP36L1*, *BTG1*, *DTX1*, and *SGK1* (18, 27–29). The abnormal transcription of these genes indicates tumorigenesis and tumor progression (**Figure 1B**) (**Table 1**).

**Table 1:** Comparison of AID’s traditional and off-target function.

	Traditional function	Off-target		
	Deamination	Mutation	Demethylation	Regulating gene transcription
Physiological processes	CSR (3, 5) SHM (3, 11)	Promoter mutation of non-Igs (12–16) Chromosome translocation between Igs and non-Igs (11)	Enhancing Ig gene transcription by converting 5mC to C in Ig genes (30, 31)	Enhancing Ig gene transcription by co-operating with RNA PolII, RPA, Spt5, etc. (26)
Pathological processes	Promoter mutation of Igs (17–29) Chromosome translocation between Igs and non-Igs (11)	Promoter mutation of non-Igs (27–29) Chromosome translocation between Igs and non-Igs (11)	DNA methylation heterogeneity (32, 33)	Activating or inhibiting the transcription of oncogenes (33, 34)
Related diseases	B cell development	Antoimmune diseases Lymphoma	Hematological malignancies Solid tumors	Hematological malignancies Solid tumors

Numbers in parentheses indicate the references.

### 3.2 AID’s demethylation function

AID can also deaminate 5-methylcytosine (5mC), creating a T:G mismatch that is faithfully repaired by downstream enzymes in the BER repair pathway, such as thymine-DNA glycosylase (TDG), resulting in the conversion of 5mC to C without a methyl group. This process demonstrates the active demethylation function of AID (30, 31, 35). It is worth noting that AID alone cannot remove the methyl groups from 5mC. In Zebrafish, the enzyme Thymine glycosylase (MBD4) can bind to 5mC through its methylated-DNA binding domain in the structure. AID is responsible for deaminating 5mC, which is then recognized as a T (35, 36). Additionally, the DNA-damage-inducible protein 45a (GADD45a) is another vital enzyme in demethylation, as it bridges AID and MBD4 (32, 33, 36).

Another suggestion of AID;s demethyaltion is that AID maybe does not directly deaminate 5mC, but rather deaminates 5-hydroxymethylcytosine (5hmC) to 5-hydroxymethyluracil (5hmU) through AID’s ability to convert C to U. The 5hmC is a substrate generated by the ten-eleven translocation (TET) process, while the 5hmU is repaired back to C by BER system ultimately resulting in the conversion of 5mC to C (37). This process suggests an alternative mechanism for AID’s demethylation (37, 38).

However, it is reported that AID’s deamination to 5mC is not fully efficiently in vertebrate and *Escherichia coli* (39, 40). The results show that Aid^-/-^ PGCs attain low levels of methylation compared with ES cells and somatic tissues, indicating that demethylation still occur even in the absence of AID (39). Thus, AID might fail to efficiently act on deamination of 5mC, other factors would take part in DNA demethylation process (39). In a comparison of the deamination among two members of human APOBEC3 family and AID in *Escherichia coli*, it is found that APOBEC3A but not AID and APOBEC3G can deaminate 5mC efficiently, resulting the conversion of C to T. AID could only deaminate 5mC weakly because the 5-methyl group fits poorly in its DNA binding pocket (41). An experiment using purified AID to study AID’s demethylation shows that its activity on 5mC reduces relative to its canonical substrate cytosine, even with no detectable deamination of 5hmC. The reactivity of a series of modified substrates indicates that steric bulk for cytosine deamination is one intrinsic barrier to AID’s demethylation in DNA (42). In fact, this discussion about steric bulk is consistent with the concept of AID’s accessibility.

Several studies have shown that AID plays a role in the reprogramming of genomic DNA methylation (43, 44). Gene promoters targeted by AID exhibit abnormally low methylation levels, indicating high activation of these genes (37, 45). AID mediated DNA methylation heterogeneity is an important aspect of heterogeneity in carcinogenesis (**Figure 1B**) (**Table 1**).

### 3.3 AID’s alternative function in regulating gene transcription

AID is also expressed in non-B cells, which means that it has the potential to regulate genes in these cells. Chronic inflammation is a well-known risk factor for cancer, with the continual production of pro-inflammatory cytokines being thought to contribute to the development of tumors (46). Recent studies have shown that tumor necrosis factor a (TNFα) triggers abnormal AID expression via nuclear factor-κB (NF-κB) in certain inflammation-related cancers such as helicobacter pylori-associated gastric cancer and colitis-associated colon cancers (34, 47, 48). Human cancers have been found with ectopic AID expression, and mutations in exons of genes related to tumors, such as TP53, have been observed in such cases (49).

The expression of ectopic AID perturbs the expression of tumor-related genes. AID, in complex with Gadd45, activates a methylated paired box gene 5 (Pax5) reporter construct and induces binding of endogenous Pax5 to AID’s promoter. This suggests that ectopic AID expression triggers an auto-activation circuit to bolster self-expression (50). It has been demonstrated that abnormal AID expression destroys normal gene expression, which also affects the regulation of the AID gene itself (50). It is believed that “accidental firing” due to leaky expression can auto-activate AID, thus ensuring robust AID expression (50). The positive feedback loop of abnormal AID expression enhances its off-target function in cancer-associated gene expression, leading to oncogene promoter mutations and chromosome translocations.

Studies on AID have traditionally focused on its deamination to cytidine in CSR and SHM. However, recent research has extended its off-target function beyond Ig-based systems, revealing its epigenetic modification capabilities through demethylation processes in the genome. Two new studies have also shown that AID regulates gene expression as a transcription cofactor, demonstrating a delicate Yin-Yang balance in its action towards cancer-associated genes. Interestingly, AID can act as a DNA demethylation factor when interacting with TET2, and as an enzyme maintaining DNA methylation when interacting with DNA methyltransferase 1 (DNMT1) (37, 51). Therefore, it is likely that AID acts as a cofactor in gene transcription by interacting with other factors. AID could interact with SPT5, which is needed to induce phosphorylation of ser5 on the CTD to restart RNA PolII after pausing. This interaction among AID, RNA PolII and Spt5 is well verified to contribute to AID targeting to transcription (52). However, Wang et al. reveal that AID binds to many proteins non-specifically, as it is a highly charged molecule (38, 53). These might suggest that AID could artificially interacted with many co-factors due to AID’s surface charge, but AID binding to RNA PolII and Spt5 are likely the only real ones (54, 55). Currently, there is no complete definition of AID’s negative or positive role as a gene transcription cofactor. AID can both activate and silence gene expression through positive or negative mechanisms, suggesting it acts as a two-sided sword that balances the function of some oncogenes by inhibiting or activating transcription. Although the exact mechanism by which AID regulates gene expression is still unknown, these two studies provide valuable insights on exploring AID’s gene regulatory network.

## 4 Keeping AID in line

Due to its mutative capacity on DNA, AID poses a threat to genomic stability. Therefore, strict regulation is necessary to prevent AID’s “off-target” from predisposing cells to malignant transformation. AID is regulated at various levels.

Firstly, limiting the off-target effects of AID could be achieved by modulating its accessibility to ssDNA. Through a combination of computer simulations and functional experiments, King et al. have uncovered a way to regulate AID activity by altering its tertiary structure. By comparing AID’s structure with and without DNA binding, and making reference to APOBEC’s tertiary structure, the researchers were able to identify functional and non-functional conformations of AID. They assessed the role of catalytic pocket accessibility and DNA binding in deamination catalysis and concluded that AID activity can be regulated by modifying the accessibility of the catalytic pocket and the DNA binding ability in its tertiary structure (53).

Secondly, AID transcription is controlled using transcription factors (TF) that bind to enhancer or repressor regions. For instance, E2F and c-Myb bind to the repressive region on intron 1, limiting AID’s transcription. Conversely, various TFs bind to different regulatory regions to enhance AID transcription (56). Furthermore, miRNAs like miR-155 and miR-181b suppress AID expression by binding to the 3’UTR of its mRNA (57, 58) (**Figure 1C**). Secondly, the AID protein has a structural nuclear localization signal (NLS) and nuclear export signal (NES) (**Figure 1A**). Through these, AID is actively shuttled between the nucleus and cytoplasm, thus limiting its possible access to DNA (59–62) (**Figure 1D**). Thirdly, PKA is recruited to the Ig locus upon antigenic stimulation, where it phosphorylates AID at S38. This allows AID to interact with RPA and APE1, critical for stabilizing the substrate and generating DNA break nicks. This post-translational modification has the most significant impact on AID activity (**Figure 1B**) (63). Lastly, ubiquitination (Ub) and proteasomal degradation of nuclear AID restrict its possible access to DNA, thus limiting AID-mediated transcription (**Figure 1B**) (64).

Thirdly, AID is involved in hormone signaling pathways that regulate gene expression. It has been reported that (i) the estrogen-bound estrogen receptor directly activates AID expression, or estrogen activates the HOXC4/HoxC4 promoter and HoxC4 expression, thereby potentiates the induction of AID expression (65); and (ii) progesterone, which is known to counteract estrogen stimulation, can inhibit the expression of AID, also through direct binding to AID’s promoter (**Figure 1E**) (66).

In brief, AID activity could be modified by regulating AID’s functional conformations in its tertiary structure. Moreover, the AICDA locus is highly regulated at the transcriptional level by several transcription factors, including NF-κB, STAT6, HoxC4, Pax5, E proteins, and Id proteins, as well as via the IL-4 and CD40 ligation pathway. Additionally, *AICDA* expression is regulated by miRNAs such as miRNA-155 and miRNA-181b, which bind to the 3’-UTR of the AICDA locus and induce mRNA degradation. Hormone signaling pathways also play a role, as estrogen and progesterone directly target the AID promoter. Post-transcriptionally, AID can be regulated by phosphorylation or ubiquitination pathways (**Figure 1**).

## 5 AID and autoimmune diseases

AID is expressed by activated B cells, particularly in the germinal centers (GCs) of peripheral lymphoid organs (5). During the humoral immune response to inflammation, antigen stimulation promotes GC formation (67). Prior to the GC reaction, in response to antigen challenge, AID levels elevate and induce CSR in the constant region of Igs to generate different types of antibodies except IgM during GC formation or even in B cells before they differentiate into GC B cells or plasmablasts (67, 68). Subsequently, AID-mediated SHM in the variable region of Igs enhances Ig diversity. Next, GC B cells in the bright zone of GCs are selected based on the antigen-antibody affinity presented by follicular dendritic cells (FDCs) and CD4^+^ Follicular T helper cells (TFH). Low-affinity antibody B cells undergo apoptosis or re-enter the dark and bright zones of GC to undergo a new round of SHM and CSR, while high-affinity antibody B cells migrate out of GCs, differentiate into plasma cells and memory B cells, and are reactivated when re-challenged by antigens (69). This GC reaction is crucial for generating high-affinity antibodies, which in turn induces robust humoral immunity.

Individuals who are unable to undergo CSR may develop hyper-IgM (HIGM) syndromes. HIGM1 is caused by a genetic defect in the CD40L expressed on T cells (70, 71); HIGM2 is caused by mutations in the *AICDA* gene (2); HIGM3 is caused by mutations in the CD40 gene (72); HIGM4 is due to a defect in B lymphocyte-intrinsic selective deficiency in Ig CSR (73); and HIGM5 is caused by mutations in the UNG gene, which hampers the process of DNA repair pathway in CSR and SHM (74). Collectively, these suggest that AID plays a role in inducing B cell tolerance. This is evidenced by the fact that HIGM patients who have high levels of serum anti-nuclear IgM antibodies are prone to developing autoimmune diseases (75, 76). These excessive anti-nuclear IgM antibodies are caused by AID’s inability to induce CSR of IgM to other Igs such as IgG, IgE, IgD, and IgA.

Furthermore, systemic lupus erythematosus (SLE), a typical autoimmune disease, is characterized by damage to multiple organs and tissues due to the production of a large number of pathogenic autoantibodies and immune complexes (77, 78). These autoantibodies are reported to be mutated or class-switched Igs (77, 78). In mice treated with 2,6,10,14-tetramethylpentadecane (TMPD) and MRL/faslpr/lpr mice, enhanced expression of AID is found in ectopic lymphoid tissue, besides the spleen, lymph nodes, and Peyer’s patches. AID causes abnormal DNA lesions, dysregulated CSR and SHM, ultimately leading to the production of pathogenic autoantibodies such as high-affinity anti-dsDNA IgG antibodies (77, 78). Therefore, maintaining a normal level of AID in canonical lymphoid structures with GC formation is vital for traditional CSR and SHM, while aberrant AID expression in ectopic lymphoid tissue results in the generation of pathogenic autoantibodies, causing autoimmune diseases such as SLE.

## 6 AID and hematological neoplasm

### 6.1 AID’s mutation in hematological neoplasm

To minimize the risk of off-target mutations, it is crucial to tightly control the activity of AID. If AID levels are abnormal, it can lead to chromosomal translocations and the development of B cell malignancies (79). Recent studies have shed light on the mechanisms by which AID targets non-Ig genes. When AID is inappropriately expressed, it acts as a DNA mutator, contributing to the development of lymphomas (79).

AID induces point mutations and chromosome translocations are responsible for most malignancies (79). AID is known to cause both WRCY and CpG/CGC breaks at BCL6 in GC B-cells, resulting in Ig-BCL6 and non-Ig-BCL6 rearrangements (80). Clinical gene rearrangement studies in diffuse large B cell lymphoma (DLBCL) cases have shown that patients with DLBCL carry abundant MYC, BCL2, or BCL6 rearrangements (70). Whole-exome sequencing has implicated that AID-caused mutated genes are related to the pathobiology of DLBCL, including *BCL6*, *PIM1*, *c-MYC*, *RHOH*, *PAX5*, *BCL2*, *MYD88*, *CARD11*, *EZH2*, *CREBBP*, etc. (51, 79–81). Therefore, it is crucial to tightly control AID activity to minimize the risk of off-target mutations and chromosome translocations.

Studies have shown that AID plays a crucial role in the development of mature B-cell lymphomas in Eμ-c-MYC transgenic mice. However, in another study, AID transgenic mice developed T-cell lymphomas but not B-cell lymphomas (28). Furthermore, overexpression of AID in B-cell lineage leads to the development and progression of mature B-cell malignancies, but only in mice with a TP53^-/-^ background (28). This suggests that tumor suppressor mechanisms are an important protective component of GC B-cell development. A unique role of AID in the pathogenesis of GC-derived B-cell lymphoma is demonstrated by the result that Iμ-HA BCL6 mice are protected from DLBCL development depending on an AID^-/-^ background. However, similar protection is not observed in AID-deficient λc-MYC and λc-MYC Iμ-HABCL6 mice, which develop pregerminal and postgerminal center B-cell lymphomas (28). This indicates that AID-generated lesions arise during the GC reaction, contributing to lymphomagenesis. However, it is worth noting that AID acts independently, and both BER and MMR processes U:G mismatches into strand lesions. Thus, although AID may initiate events that lead to GC-derived lymphoma, associated DNA repair pathways are likely needed to be dysfunctional in critical steps to drive mutations and chromosome translocations.

Furthermore, studies have shown that AID can promote and accumulate the same gene mutations in the TCL1 and Eμ-TCL1 mouse models, also in human chronic lymphocytic leukemia (CLL) patients. This suggests that AID promotes the aggressiveness of CLL and other B-cell neoplasms (28, 82). However, in the AID^−/−^/Eμ-TCL1 mouse model, AID is found to eliminate the progression of CLL. The secretory IgM-deficient CLL cells in the AID^−/−^/Eμ-TCL1 mouse express less transcription factor XBP1s, which depletes B cell receptor signaling, inhibiting leukemic growth and survival. This explains why non-mutated CLL is more aggressive than mutated CLL (83). In summary, AID is associated with many events, such as DNA repair pathways, that promote mutations and chromosome translocations, contributing to lymphomagenesis. However, utilizing AID’s off-target effects may be a promising way to alleviate some hematological neoplasms.

### 6.2 AID’s epigenetic role in hematological neoplasm

Both epigenetic and genetic alterations are known to contribute to the initiation and progression of cancer. Epigenetics refers to the study of heritable changes in gene expression without alterations in DNA sequences (84). These changes include DNA methylation, chromatin modifications, nucleosome positioning, and alterations in noncoding RNA profiles (85). Disruptions in these processes can lead to altered gene function and cellular neoplastic transformation (86). Epigenetic modifications usually occur at an early stage in neoplastic development and often precede genetic changes.Recent studies have reported that AID-mediated methylation diversity changes mainly contribute to the initiation, progression, and metastasis of hematological neoplasms (87). In addition to AID’s direct targeting of DNA, studies have shown that AID and TET family members regulate active DNA demethylation (37). AID and DNMT1 are also involved in maintaining DNA methylation in DLBCL (51). The direct or indirect effect of AID in generating DNA methylation diversity is the most likely mechanism for the development of hematological neoplasms.

## 7 AID and solid tumors

In addition to its well-known role in B cells, AID has also been found to play a role in inducing both CSR and SHM in fibroblasts by ectopic expression in GC-like structures through the addition of substrates (88–90). Based on this understanding of AID’s function, it is proposed that AID is involved in tumorigenesis through three mechanisms: (i) inducing promoter mutations and chromosome translocations of tumor-associated genes through AID’s mutagenic activity; (ii) mediating epigenetic modifications of tumor-associated genes through AID’s demethylation activity; and (iii) regulating the transcription of tumor-associated genes through AID’s function as a transcription cofactor.

As a gene mutator, AID induces promoter mutations and chromosome translocations mainly through AID’s deamination-mediated genome instability in cancer cells, which is critical for tumor progression (17, 19, 22). Pioneering cytogenetic studies have successfully defined recurrent chromosome changes in specific types of tumors (24), firmly establishing the central role of sequential accumulation of genetic alterations during cancer development. During carcinogenesis, cells undergo several genetic alterations that are associated with the transition from a pre-neoplastic lesion to an invasive tumor. Understanding the genetic mechanisms involved in tumorigenesis can provide new clues to predict and control tumor development. It is known that AID targets tumor-associated genes, generating multiple mutations and chromosome translocations, and these abundant genetic alterations mediate tumorigenesis in 50% patients (91).

Currently, there is limited understanding of the critical epigenetic events that contribute to tumor progression. AID’s active demethylation, which is based on its deamination to 5mC, has received significant attention. Through investigating the *in vivo* epigenetic function of AID in GC B-cells isolated from wildtype (WT) and AID-deficient (Aicda^-/-^) mice, it has been determined that the B-cell transit in GC is associated with a marked locus-specific loss of methylation and increased methylation diversity in Aicda^-/-^ animals. This indicates that AID is a key factor in epigenetic reprogramming in mouse and human B-cells (92). In tumors, the absence of AID has been shown to alter epigenetic diversity and differentially methylated cytosines (DMCs) in the genome, suggesting different gene expression profiles. This involves various aspects related to tumor progression, including genome stability, cell biological functions, and the tumor environment (93, 94). Conventionally, AID’s epigenetic modification to tumor-associated genes is related to epigenetic modification factors such as TETs, DNMTs, and HDACs (37, 51).

Furthermore, transcriptionally active regions such as enhancers and promoters are involved in determining the timing and location of gene transcription in response to intrinsic and extrinsic signals (24). These regions contain binding sites for sequence-specific transcription factors and coactivators that determine the spatiotemporal specificity of gene activity during development. Detection of AID 3D-linked targets has revealed that AID targets are not randomly distributed across the genome, but are primarily clustered within super-enhancers and regulatory clusters (24). Topological structure-determined gene accessibility forms the basis of AID targeting, as recruitment of RNA PolII, Spt5 and RPA creates an open genetic structure for gene transcription, providing the favorable conditions for AID recruitment and interaction with ssDNA (26). Previous studies have shown that AID cooperates with DNMT1 to repress BCL6 expression, while the AID-TET2 complex promotes FANCA transcription in DLBCL (37, 51). This suggests that AID has a Yin-Yang regulation in gene transcription, modulating gene activation or silencing. The reported negative regulation of AID in the process of CSR and SHM in B cells confirms that AID inactivation occurs even under the accumulation of abundant AID protein (94). This suggests that excessive AID is not necessarily harmful, and deficient AID is not necessarily beneficial. It further identifies both the positive and negative roles of AID in tumorigenesis.

**Figure 2:**
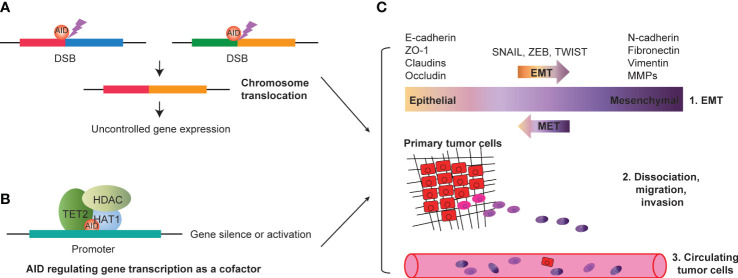
AID and progression of solid tumors. **(A)** AID mediated DSBs could induce chromosome translocation, resulting in uncontrolled tumor associated gene expression. **(B)** AID interacts with transcription factors (such as HDAC, TET2 and HAT1, etc.), and regulating gene expression as a cofactor. The gene silence or activation depends on AID interacted factors’ action. **(C)** AID mediated EMT by targeting EMT associated genes (such as E-cadherin, ZO-1, Claudins, Occludin, N-cadherin, Fibronectin, Vimentin, MMPs, etc.). AID could promote the progression of solid tumors by mediating the transcription of genes related to dissociation, migration and invasion. Finally, the primary tumor cells could conduct distal or proximal metastasis through circulating tumor cells by blood vessels. DSBs, double strand breakages; EMT, epithelial-mesenchymal transition.

In summary, the concept of off-target AID in solid tumors could be extended to encompass the following three mechanisms: (i) AID-mediated mutations that regulate gene expression through promoter mutations or chromosome translocations (**Figure 2A**); (ii) AID’s epigenetic modification that mediates gene expression regulation through demethylation or coupling with other epigenetic modification factors such as DNMTs and HDACs (**Figure 2B**); and (iii) AID may mediate gene transcription regulation as a cofactor, then promote or repress tumor-associated gene transcription, depending on the action of its cooperated factors (**Figure 2B**). All three regulation mechanisms of solid tumor-associated genes by AID may be attributed to AID’s off-target effect. First, AID mediates EMT (epithelial-mesenchymal transition) by targeting EMT-associated genes. Second, AID promotes the progression of solid tumors by mediating the transcription of genes related to dissociation, migration, and invasion. Finally, the primary tumor cells conduct distal or proximal metastasis through circulating tumor cells by blood vessels (**Figure 2C**). Revealing AID’s gene expression regulatory mechanism suggests the off-target effect of AID in the tumorigenesis of various cancers, such as colorectal cancers (34), breast cancers (91), gastric cancers (95), bladder cancers (96), lung cancers (97), melanoma (98), and others.

## 8 Conclusion: AID acts as a positive or negative factor in carcinogenesis

AID plays a crucial role in regulating SHM and CSR of Igs in the immune system. However, it can also be a dangerous enzyme when its off-target activities occur. Interestingly, AID^-/-^ mice have been found to be resistant to disease development and only generate low-affinity IgM antibodies. Patients with abnormal AID expression (such as mutations in the AID gene sequence) suffer from HIGM, characterized by elevated serum IgM and low-affinity antibodies (93). In addition, a subgroup of these patients also experiences autoimmunity with auto-reactive IgM antibodies present in their serum. These findings suggest that AID’s functions may vary under different conditions, either preventing or promoting disease development.

Additionally, the role of AID in regulating epigenetic reprogramming may extend beyond the immune system and into diseases such as neoplasms and tumors. The emergence of DMCs between germinal center B (GCB) cells and naive B (NB) cells isolated from both wild-type and Aicda^-/-^ mice suggests the epigenetic function of AID during GC formation (92). Furthermore, a separate study indicated that AID-mediated deamination of cytidine - followed by BER and MMR - can actively or passively remove DNA methylation marks, which could potentially contribute to DNA hypo-methylation and subsequent carcinogenesis (41).

A new function of AID has been identified, namely its role in the regulation of gene expression. In a breast cancer cell model, increased expression of AID facilitated the transcription of genes associated with EMT and led to enhanced invasive behavior (99). Activation of EMT is a critical step in tumorigenesis, which suggests that AID may possess oncogenic potential in cancers.

Clearly, AID is an extraordinary enzyme with a broad capacity to regulate development, the immune system, and human health. The off-target effects of AID are summarized in this review, which includes three parts: mutations of non-Igs, epigenetic modifications, and the alternative role as a cofactor. It is worth noting that the off-target effects of AID elucidated in this study do not only indicate AID’s role in carcinogenesis, but also highlight AID’s bilateral function in carcinogenesis. AID’s mutations in non-Igs can either lead to sustained activation or inhibition of cancer-associated genes. Epigenetic modifications can also result in cancer-related gene transcription or silence. The newly proposed function of AID as a cofactor further suggests the positive and negative roles of AID in regulating cancer gene expression. In summary, AID would act as a positive or negative factor in carcinogenesis. At present, major questions remain regarding how to regulate and target this essential but dangerous enzyme to specific loci to treat diseases.

## Author contributions

JJ and ZL searched for literature and wrote the manuscript. YW and LF were involved in edited the manuscript. AY supervised the project and contributed to the revision of the final manuscript. All authors contributed to the article and approved the submitted version.
